# How to know when little kidneys are in trouble: a review of current tools for diagnosing AKI in neonates

**DOI:** 10.3389/fped.2023.1270200

**Published:** 2023-11-21

**Authors:** Rebecca E. Evans, Jennifer Peterson, Jon Jin Kim, Ajit Mahaveer

**Affiliations:** ^1^ St. Mary's Neonatal Unit, Manchester University NHS Foundation Trust, Manchester, United Kingdom; ^2^ Faculty of Biology, Medicine and Health Sciences, The University of Manchester and Manchester University NHS Foundation Trust, Manchester, United Kingdom; ^3^ Department of Paediatric Nephrology, Nottingham Universities Hospitals NHS Trust, Manchester, United Kingdom

**Keywords:** neonatal renal clearance, creatinine blood, acute kidney injury, neonatal acute kidney injury, renal biomarker, acute kidney injury biomarker

## Abstract

Due to a plethora of risk factors, including prematurity, neonates are at risk for acute kidney injury (AKI) and, once established, AKI is associated with poor outcomes. The most widely used AKI biomarker is creatinine, despite research demonstrating creatinine to be a suboptimal tool for diagnosing neonatal AKI. This article uses an amalgamated case study to illustrate the inadequacies of creatinine for detection of preterm AKI and to present a range of novel AKI biomarkers relevant to the neonatal population. Clinical evaluation of novel AKI biomarkers is needed to improve precision and rapidity of AKI management in neonates.

## Introduction

Acute kidney injury (AKI) is common in the neonatal population due to a number of important and common risk factors. Nephron development is not complete until 32–36 weeks gestation (1), and research demonstrates overall reduced nephron numbers in preterm infants compared to term infants (2). This likely predisposes them to long-term damage from AKI. Growth restricted infants have also been found to have a higher risk of AKI compared to infants of a normal size for their gestation. A small study of low birthweight (LBW) infants found that 87% of very LBW [VLBW, i.e., birth weight <1,500 grams(g)] infants are exposed to at least one nephrotoxic medication (3). They also found that the average VLBW infant is exposed to almost two weeks of nephrotoxins, including commonly used medications, e.g., aminoglycosides for sepsis screens and ibuprofen for patent ductus arteriosus (PDA) closure (3). Other common risk factors for AKI in newborn infants include perinatal asphyxia which is associated with AKI in 30%–56% of cases (4), and congenital heart disease (5).

## Case illustration^1^

You admit a 23 + 5 week gestational age female infant (Infant A) to the neonatal intensive case unit (NICU), weighing 520 g. The mother received a full course of steroids (two doses of dexamethasone intramuscularly 24 hours apart) and magnesium sulphate antenatally. Infant A is intubated and ventilated at birth and receives two doses of Curosurf™ over the first 24 hours. She is stable on conventional ventilation, her sepsis screen was negative and antibiotics were stopped at 36 hours. She is normothermic in 90% humidity. Due to her prematurity and fragile skin, she is at risk of excessive insensible losses. The medical team attempts to compensate for this with administration of increased fluid volumes (up to 180 ml/kg/day), whilst trying to balance risks of oedema and hyponatraemia.

**Figure 1:**
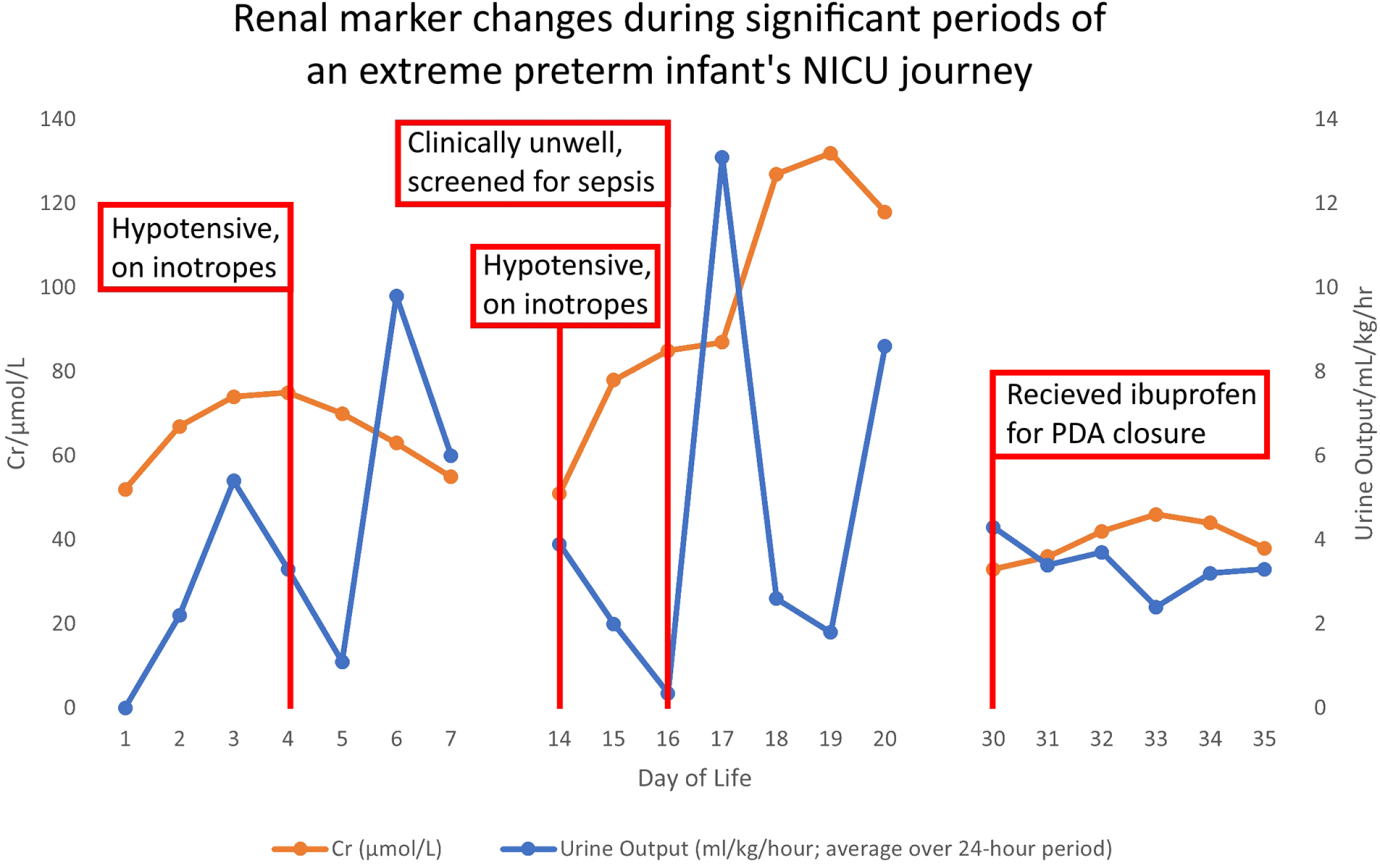
Demonstrates the changes in creatinine and urine output plotted against days of life, to illustrate case points 1–2.

In the first 48 hours, infant A had significantly elevated sodium and urea, whilst serum creatinine (SCr) remained within normal limits (Figure 1) (Supplementary Appendix C). She became hypotensive on day 4 of life requiring inotropic support until day 6.

## Grading AKI

Given the physiologically significant events over the first week, Infant A's medical team may anticipate risk of AKI. There are two paediatric AKI grading systems, both relying on SCr; The Kidney Disease: Improving Global Outcomes (KDIGO) and paediatric risk of renal dysfunction, injury to the kidney, failure of kidney function, loss of kidney function and end-stage renal disease (pRIFLE). KDIGO criteria uses SCr and urine output (UO) to diagnose and grade AKI severity. Strategies using SCr and UO to diagnose AKI were initially introduced in adults, although KDIGO is now validated in paediatric populations (6) and a modified criteria is available for neonates (5). (Supplementary Appendix A).

pRIFLE stratifies risk from AKI in paediatric populations using UO and SCr clearance (7). There is a modified neonatal version (nRIFLE) with altered UO limits but no defined SCr values (8) (Supplementary Appendix B).

Both criterion rely on SCr to grade severity of kidney dysfunction which is not an accurate biomarker of AKI in neonates, and the nRIFLE solely relies on UO.

## Returning to case illustration

The first week of life is physiologically turbulent for Infant A. Her UO decreases from day 3, coinciding with hypotension requiring inotropes. Inadequate blood supply to the kidneys reduces the glomerular blood flow. The reduction in UO with hypotension would likely indicate an episode of injury to the kidneys. However, using current, standard, SCr-based criteria, the SCr levels for Infant A remain within acceptable parameters. Neither KDIGO, pRIFLE or nRIFLE would classify her as having had an AKI.

## Why worry about AKI?

Blood based renal markers are used to provide objective evidence of AKI, e.g., SCr increases with decreasing kidney function. Whereas earlier physiological signs of AKI, such as altered electrolytes and reduced UO, are subtle and easily missed. There are currently no other clinically validated markers of kidney function in neonates though some studies have used cystatin C (Table 1). As we cannot aptly identify early AKI in neonates, we cannot implement management strategies early; this likely leads to more severe illness and co-morbidities (17).

**Table 1:** Urinary and serum biomarkers of acute kidney injury with background information and advantages and disadvantages of their use in neonates.

Urinary biomarker	Site of expression	Background	Advantages	Disadvantages	Cost of test (£)
Interleukin-18 (IL-18)	Released by proximal tubule.	Pro-inflammatory cytokine, released in response to tubular injury.	Significantly higher in non-septic critically ill infants with acute kidney injury (9). Normal value doesn’t change with increasing renal maturity.	Can be deranged with sepsis.	£30
Kidney injury molecule-1 (KIM-1)	Shed from proximal tubule cells.	Type-1 transmembrane glycoprotein not normally detected in urine. Secreted into urine after ischaemic or toxic injury.	One large scale study has demonstrated significantly higher levels of urinary KIM-1 in asphyxiated newborns with acute kidney injury (10).	Studies are inconclusive, some have shown no statistical significance in levels associated with acute kidney injury.	£30
Neutrophil gelatinase associated lipocalin (NGAL)	Filtered by the glomerulus and rapidly reabsorbed by the proximal tubule.	Ubiquitous 25-kDA protein covalently bound to gelatinase from human neutrophils, member of lipocalin superfamily and also has immunological functions.	Could be early predictive marker of AKI after cardiopulmonary bypass (11).	Deranged in sepsis and inflammation.	£24
Cystatin C (CysC)	Freely filtered through the glomerular membrane and completely reabsorbed and degraded by the proximal tubule.	Low molecular weight protein in protease inhibitor class.	More sensitive than serum CysC and can predict acute kidney injury in neonates with low APGAR scores (12). Early sensitive method for detecting acute kidney injury.	Levels decrease over first few days of life and some studies have found levels only positively correlate with asphyxia and acute kidney injury on day of life1–3.	£4
Serum Biomarker	Site of expression	Background	Advantages	Disadvantages	Cost of test (£)
NGAL	Filtered by the glomerulus and rapidly reabsorbed by the proximal tubule.	Ubiquitous 25-kDA protein covalently bound to gelatinase from human neutrophils, member of lipocalin superfamily and also has immunological functions.	Shown to be significantly higher in neonates with AKI at 2 and 4 h life (13).	Also deranged in inflammation and sepsis.	£24
CysC	Freely filtered through the glomerular membrane and completely reabsorbed and degraded by the proximal tubule.	Low molecular weight protein in protease inhibitor class.	Shown to be significantly higher in severely asphyxiated neonates and can be predictive of acute kidney injury (14). Does not cross the placenta.	More studies required for cut-off values.	£4
*Β*eta-trace protein (BTP)	Freely filtered through the glomerulus.	23–29 kDa enzyme.	Concentration is independent of gestational age, more sensitive marker of acute kidney injury than creatinine (15).	Levels are different between genders. Can also be a marker of cardiovascular risk so may be deranged if myocardial damage secondary to perinatal asphyxia.	£42
Β-2-microglobulin (B2mG)	Freely filtered through glomerular membrane and catabolised in tubules.	Belongs to low molecular weight proteins.	Significantly associated with asphyxia and acute kidney injury (16).	Also deranged in respiratory distress syndrome. Low sensitivity.	£3.69
Symmetrical dimethyl arginine/Aymmetrical dimethyl arginine (SDMA/ADMA)	Filtered through glomerulus.	Metabolites of the amino acid L-arginine.	Not dependent on muscle mass or maternal renal function.	Data from adult and veterinary patients; not validated in infants.	£15

Predicting onset of AKI and altering fluid management promptly could help reduce complications. Acute, significant changes in electrolytes and blood pressure contribute to a risk of intraventricular haemorrhage (IVH) in preterm infants (18). There are also studies that suggest large volumes of intravenous fluid can contribute to the ductus arteriosus remaining patent in the first few days of life (19). A haemodynamically significant PDA has the potential to increase the risk of AKI through systemic steal (20). The medical management of a PDA can further contribute to AKI through administration of nephrotoxic medications, such as ibuprofen (21).

Long term complications may occur after resolution of AKI. Studies show that repeated or severe AKI can result in chronic kidney disease (CKD) (22).

In 2014, the Assessment of Worldwide Acute Kidney Injury Epidemiology in Neonates (AWAKEN) study evaluated the association between AKI and mortality in critically ill neonates with gestational ages of 22–40 weeks. This demonstrated a 4-fold higher mortality risk and longer hospital stay for infants with AKI compared to those without (23). It also showed that preterm infants (22–29 weeks gestation) were more likely to have repeated AKI after the first week of life (23).

## Case illustration continued

On day 14, Infant A has increasingly frequent bradycardic episodes associated with desaturation. She is started on intravenous cefotaxime and vancomycin, her C-Reactive Protein increases to 46 mg/dl and blood cultures grow *Staphylococcus Capitis* and *Hominis*. She is treated for line sepsis and, due to a septic ileus, is nil by mouth and commenced on intravenous fluids and then total parenteral nutrition (TPN) via a newly inserted central line. After 48 hours her UO drastically decreases, and she becomes virtually anuric (Figure 1) (Supplementary Appendix C). Her Vancomycin level rises to >20 micrograms/ml and Vancomycin is held. The SCr level rises modestly from 51 μmol/L to 85 μmol/L during this period (Figure 1, Day 14–16) (Supplementary Appendix C), whilst Infant A becomes hypotensive with a mean arterial BP of 20 mmHg and a rise in lactate to 4.0 mmol/L. She receives a 10 ml/kg fluid bolus and recommences inotropes.

Between day 16–19 (Figure 1) (Supplementary Appendix C), there is a delayed SCr rise, secondary to this sepsis-induced AKI. Infant A's SCr peaks on day 19, 72 hours after she has improved clinically and her UO has normalised.

## Why is SCr not peaking sooner?

SCr is an easy and practical method of measuring the glomerular filtration rate (GFR) in adult populations. However, there are many limitations to its use in the neonatal cohort. In the first 72 hours of life, SCr in the newborn reflects maternal levels. In a neonate with normal renal function, the SCr will decrease until reaching a baseline which can take 1–2 weeks in a full-term baby, and up to 6 weeks in preterms (24). Furthermore, SCr levels are based on muscle mass, which is low in infants (12). This renders the cut-off levels used in adult populations difficult to extrapolate to infants, and inaccurate when used in neonatal populations.

## The problem with urine output

Declining UO can be a late sign of AKI and is difficult to monitor. UO is usually measured in neonates by weighing nappies to measure the volume of urine. However, often nappies also contain stool, and can leak, rendering this measurement inaccurate. Furthermore, neonates have higher total body water content and immature tubular development and therefore have a greater baseline UO compared to children and adults. A study that reviewed the UO cut-offs used for the pRIFLE criteria showed these to be too low and therefore diagnosing AKI too late in the neonatal population. They recommended that a UO threshold of <1.5 ml/kg/h be used to improve capacity to detect AKI (25).

## Case illustration continued

At one month, Infant A continues to have a significant oxygen requirement. Echocardiogram shows a haemodynamically significant PDA. The baby's renal function appears to have returned to baseline and she has a normal UO so it is felt to be safe to treat with ibuprofen. Whilst she is receiving ibuprofen there is a subtle increase in SCr and a drop in UO, but neither reach the threshold to diagnose AKI. Therefore it is assumed it is safe to continue ibuprofen.

## Nephrotoxic medications

Ibuprofen is known to be nephrotoxic and clinical studies have demonstrated that ibuprofen causes AKI when used for PDA closure. One study shows that the declining use of prophylactic non-steroidal anti-inflammatory drugs has been associated with reduced rates of AKI in preterms (26). There are subtle changes in SCr and UO in Case Illustration 3 which likely indicate an undiagnosed AKI. However, these changes are not enough to stop ibuprofen and its continuation has potential to cause more damage. If not detected and managed acutely (for example, by withholding nephrotoxic medications and altering fluid intake), the consequence could be CKD in later life. This has been demonstrated by several studies reviewing long term impacts of AKI in infants and children. One prospective cohort study published in 2022 found that one third of children who had been diagnosed with KDIGO stage 1 to 2 AKI as a neonate had at least one sign of long-term kidney dysfunction. These included: hypertension, proteinuria and hyperfiltration (27). With these proven adverse consequences to AKI in early life, it becomes even more important to detect and treat AKI before it causes further damage.

## Alternative biomarkers of AKI

Given the diagnostic quandary neonatal clinicians face there have been many studies in recent years looking into alternative biomarkers that could be used to diagnose AKI at onset or predict AKI.

## Novel biomarkers

Many researched novel biomarkers are urine based with research conducted in term asphyxiated infants or older infants undergoing cardiac surgery. Obtaining urine samples is problematic in neonatal patients and there are few reliable methods for collection. Cotton wool ball collection is challenging as neonates will frequently have passed stool in the nappy with the urine. In-out catheterisation should be avoided where possible due to the risk of infection. Urine collection with a bag is complicated by the risk of skin irritation or damage to newly delivered/premature infants. Therefore, urinary biomarkers have limited utility in neonates.

Of the urinary biomarkers that have been studied, the most promising seem to be interleukin-18 (IL-18), kidney injury molecule-1 (KIM-1), beta2-microglobulin (B2mG), neutrophil gelatinase associated lipocalin (NGAL), and cystatin C (CysC).

IL-18 is an interleukin-1 family pro-inflammatory cytokine produced by macrophages and monocytes. It is an inflammatory mediator and levels are increased in endogenous inflammatory processes such as sepsis. IL-18 production is also upregulated in response to ischaemia in different organs (28). Therefore, IL-18 is not reliable as a biomarker of AKI in neonates given how its levels can be influenced by many inflammatory processes.

KIM-1 is a type 1 transmembrane glycoprotein which acts as an early biomarker for renal tubular injury and has low levels of expression in healthy renal tissue. KIM-1 is rarely expressed in other organs so it can be specific for renal damage and studies have found urinary levels to rise significantly within one hour following tubular injury. Animal studies have shown levels to increase significantly with ischaemic renal damage, and drug-induced AKI (29). However, studies have demonstrated that co-morbidities such as hypertension and cerebral ischaemia, as well as inflammatory processes, may alter concentrations of KIM-1 (28).

B2mG is a single-chain low molecular weight peptide which exists as part of the light chain portion of Major Histocompatibility Complex Class I molecules on cell membranes. B2mG is mainly produced through cell membrane turnover, then filtered through the glomeruli, and reabsorbed and catabolised by cells in the renal proximal tubule, leaving very small amounts of B2mG excreted in the urine. Therefore, raised urinary B2mG suggests tubular dysfunction. A small study in term neonates found urinary B2mG levels to be significantly raised in infants with AKI and asphyxia (30). However there are limited studies of B2mG in neonates with little data on how its levels may differ depending on weight, gestational age and co-existing inflammatory processes.

NGAL and CysC are the most commonly studied biomarkers of AKI and seem to be the most promising but even these have drawbacks.

NGAL, a protein expressed in many cells including neutrophils, epithelial cells, and excretory systems including the kidneys, is a member of the lipocalin superfamily. It is rapidly eliminated from circulation with a half-life of 10–20 min (31). It is reabsorbed by the proximal renal tubules, and in AKI, released by injured distal tubules with levels rising within hours of renal insult (28). NGAL has been shown to be associated with renal regeneration and repair following asphyxia in mice (31). Levels have been found to increase within 3 hours of renal insult and peak at around 6–12 hours (32). Neonatal studies have shown NGAL to be a sensitive marker of AKI and increasing urinary levels reflect tubular and epithelial injury. However, these same studies have also found NGAL to increase with sepsis, and elevated serum CRP correlates with increased serum and urinary NGAL. There is also a negative correlation between birth weight, gestational age and urinary NGAL, meaning NGAL levels are higher in preterm, LBW infants. This needs further research to find appropriate cut off values based on gestational age and birth weight (33).

CysC is a proteinase inhibitor involved in intracellular catabolism of proteins and peptides. It is freely filtered by the glomeruli and broken down by the proximal tubules. CysC cannot cross the placenta and therefore solely represents neonatal levels. Serum CysC should increase and be predictive of AKI earlier than urinary in the course of tubular injury (34). Because of this, urine CysC is less useful than serum, and would not be helpful in predicting onset of AKI. This was demonstrated in a meta-analysis comparing serum and urine CysC levels in children with AKI by Nakhjavan-Shahraki et al. This found that whilst serum CysC was significantly higher in AKI patients compared to non-AKI, urine CysC took longer to rise secondary to AKI and was not significantly associated with AKI. This may be because, for urine CysC levels to increase there must be tubular injury, but in many patients with stage 1 AKI tubular injury has not yet occurred (35).

With these weaknesses, and the difficulty of obtaining a urine sample, clinicians are likely to find serum biomarkers preferable (Table 1).

 Unfortunately most of these novel serum biomarkers also have drawbacks and studies of their diagnostic utility are limited in preterm and LBW neonates. Of the serum biomarkers of AKI available, the most researched ones include: NGAL, CysC, Beta-Trace Protein (BTP), B2mG and SDMA/ADMA.

Pejovic B et al. demonstrated that serum NGAL positively correlates with periods of asphyxia in newborn infants, and can predict onset of AKI from 2 hours of age (13). However, as with urinary NGAL, serum NGAL is not specific to renal damage and its concentration is increased with systemic infections (36), a common affliction in neonates. This renders NGAL an unreliable marker of AKI in the neonatal population.

Elmas et al. determined that serum CysC is highly specific for diagnosing AKI in neonates on day 3 of life with a cut off value of 1.62 mg/L, however, with a low sensitivity (16%) (37). A meta-analysis by Yang H et al., demonstrated that serum CysC rises earlier than SCr during the early stages of AKI, but concluded larger, wider scale studies are required in the neonatal population to confirm the utility of CysC, and to determine cut off values (38).

BTP, also known as prostaglandin D2 synthase, is an endogenous marker of GFR. It is a low molecular weight protein which is freely filtered through the glomerulus and mostly renally excreted. The half life is 1.2 hours, much shorter than that of SCr (3.8 hours), and it has been postulated it would make a more sensitive marker of AKI. Indeed, in a study of 75 children aged between 2 and 19 years, BTP was significantly more sensitive at diagnosing AKI than SCr, but not more sensitive than CysC (39). Further studies have demonstrated that triglycerides and corticosteroids may independently affect levels of serum BTP (40), and this could cause difficulties interpreting levels in neonates receiving TPN/steroids.

Serum B2mG has also been postulated to be a good predictor of GFR. However, its serum concentration can be influenced by acute-phase reactants and therefore it is difficult to interpret in the context of sepsis or other inflammatory disorders (39).

Lastly, Symmetrical Dimethyl Arginine (SDMA) and Asymmetrical Dimethyl Arginine (ADMA) are metabolites of arginine. Arginine is metabolised within the mitochondria and SDMA/ADMA are released into the cytoplasm and transported into the circulation. More than 90% of SDMA is renally excreted after being filtered by the glomerulus, and therefore serum levels correlate well with GFR. Levels of SDMA are not influenced by extrarenal factors such as body mass, or inflammation, and studies have shown that serum SDMA increases with renal impairment and progressive nephron loss in humans and in animals with CKD (41). Studies have shown SDMA levels in humans to be strongly correlated with GFR and to be more significantly associated with AKI than SCr (42).

## Discussions

AKI is a common medical problem encountered in NICU, associated with significant mortality. Current methods used for diagnosing AKI are not useful for identifying renal injury in neonates at the time of onset. Better diagnostic tools are required in order to predict onset of AKI and therefore alter management earlier to prevent complications and mortality.

There are novel biomarkers, both urinary and serum, but none are yet fully validated in a neonatal cohort and each one has its own disadvantages. Current studies have demonstrated that serum CysC may be the most accurate biomarker of AKI in neonates but more research is needed for trends and cut off values.

SDMA looks to be a very promising biomarker of AKI that is not influenced by inflammation or muscle mass and strongly correlates with estimated GFR. Its utility at diagnosing AKI in neonates needs to be evaluated in clinical studies, as, to date, it has mainly been studied in adults and animals.

More studies are needed in this area so that we can better diagnose and manage AKI and aim to improve outcomes for neonatal patients.

## Footnotes

^1^The case illustrations in this article have been derived from a combination of clinical neonatal cases. This has been done to preserve patient anonymity, whilst illustrating the difficulties that detection and management of neonatal AKI poses for clinicians.
