# Supplementary material for: How to know when little kidneys are in trouble: a review of current tools for diagnosing AKI in neonates
Source: Front Pediatr. 2023 Nov 21;11:1270200. doi: 10.3389/fped.2023.1270200 (PMC10702944; doi:10.3389/fped.2023.1270200)
Supplement: Supplementary file 1 [file Datasheet1.pdf]

Appendix A: Definition and Classification of acute kidney injury according to the Kidney Disease: Improving Global Outcomes (KDIGO) criteria, adapted for use in paediatrics, 2012. [5] **Modified for use in neonates 2015.** [6]

	Paediatric		Neonatal	
Stage	Serum Creatinine	Urine Output	Serum Creatinine	Urine Output
1	1.5-1.9 X baseline OR ≥0.3mg/dL increase within 48 hours	<0.5ml/kg/hr for 6-12 hours	≥0.3mg/dL rise within 48 hours or ≥1.5-1.9 X rise from baseline within 7 days	≤ 1ml/kg/hr for 24 hours
2	2-2.9 X baseline	<0.5ml/kg/hr for ≥ 12 hours	2-2.9 X baseline	≤0.5ml/kg/hr for 24 hours
3	3 X baseline OR increase in serum creatinine to ≥4mg/dL OR initiation of Renal Replacement Therapy (RRT) OR in patients < 18years, decreased estimated glomerular filtration rate < 35ml/min per 1.73m ^{2}	<0.3ml/kg/hr for ≥ 24 hours OR anuria for ≥ 12 hours	≥ 3X rise from baseline or serum creatinine ≥ 2.5 mg/dL or RRT initiation	≤0.3ml/kg/hr for 24 hours

Appendix B: Definition and Classification of acute kidney injury according to paediatric risk of renal dysfunction, injury to the kidney, failure of kidney function, loss of kidney function and end-stage renal disease criteria (pRIFLE), formulated for use in paediatrics, 2007. [7] **Modified for use in neonates, 2013.** [8]

	pRIFLE		nRIFLE	
Category	Estimated Creatinine Clearance	Urine Output	Estimated Creatinine Clearance	Urine Output
Risk (R)	Decrease by 25%	<0.5ml/kg/hr for 8 hours	N/A	<1.5ml/kg/hr for 24 hours
Injury (I)	Decrease by 50%	< 0.5ml/kg/hr for 16 hours	N/A	<1 ml/kg/hr for 24 hours
Failure (F)	Decrease by 75% or <35ml/min/1.73m ^{2}	<0.3ml/kg/hr for 24 hours or anuric for 12 hours	N/A	<0.7 ml/kg/hr for 24 hours or anuric for 12 hours
Loss (L)	Loss of renal function > 4 weeks		N/A	

End-Stage (E)	End Stage Renal Disease		N/A	

Appendix C: Summary of Case:

	Day of Life	Clinical descriptor	Na+ (mmol/L)	Ur (mmol/L)	Cr (µmol/L)	Urine Output (ml/kg/hour; average over 24-hour period)
**Week One**	1	Born at 23 weeks gestation. Birth weight 520 grams. Intubated and ventilated at birth. Given surfactant and admitted to neonatal intensive care. C-reactive protein low and blood culture negative. Received inotropic support due to hypotension day 4 – 6.	145	5.8	54	0
	2		155	11.9	67	2.2
	3		153	14.6	74	5.4
	4		149	11.9	75	3.3
	5		141	9.3	70	1.1
	6		132	7.6	63	9.8
	7		139	4.5	55	6.0
	14	Septic episode. C-Reactive protein peaked at	140	4.2	51	3.9
	15		138	6.2	78	2.0
	16		152	6.7	85	0.35

**Week Two**	17	46 µmol/L on day 16 of life. Blood culture *Staph capitis* and *homonis*. Then cleared. Required inotropes day 15 to 17.	147	5.6	87	13.1
	18		147	6.4	127	2.6
	19		153	6.3	132	1.8
	20		148	4.3	118	8.6
**One Month**	30	Haemodynamically significant patent ductus arteriosus. Received ibuprofen treatment for 72 hours (Day 31 – 33). Post-treatment ECHO showed ductus closed.	135	4.7	33	4.3
	31		137	2.9	36	3.4
	32		138	2.7	42	3.7
	33		142	2.2	46	2.4
	34		138	2.5	44	3.2
	35		137	4.5	38	3.3
**Discharge**	140	Discharged home. Now weighing >2.5kg. Chronic lung disease going home on nasal cannulae oxygen. Feeding well	142	4.2	15	No longer quantified. Passing urine recorded in notes.

		combination of bottle and nasogastric tube. Small intraventricular haemorrhages. Handling appropriately.				
